# Reply to Nayak, P.K. Comment on “Samulewski et al. Magnetite Synthesis in the Presence of Cyanide or Thiocyanate under Prebiotic Chemistry Conditions. *Life* 2020, *10*, 34”

**DOI:** 10.3390/life11121416

**Published:** 2021-12-17

**Authors:** Rafael Block Samulewski, Flávio F. Ivashita, Andrea Paesano, Dimas Augusto Morozin Zaia

**Affiliations:** 1 Departamento de Química, Universidade Estadual de Londrina, Londrina CEP 86057-970, PR, Brazil; blockeness@gmail.com; 2 Departamento de Física-CCE, Universidade Estadual de Maringá, Maringá 87020-900, PR, Brazil; fivashita@gmail.com (F.F.I.); paesano@wnet.com.br (A.P.J.)

We have considered the criticisms raised by Pranaba K. Nayak [1] regarding the Mössbauer analysis presented in our paper “Magnetite Synthesis in the Presence of Cyanide or Thiocyanate under Prebiotic Chemistry Conditions” [2] and, in our opinion, the issues addressed make sense. However, we do not believe that any of them implied mistakes regarding the final conclusions of the paper.

First of all, we recognize that the Mössbauer spectra of the MG4P, MG4CN, and MG4SC samples should be fitted considering three sextets and a doublet (thus performing 20 lines and not 18, as pointed out by Nayak). In this sense, we believe that the complainant is correct. Therefore, new fits were performed—for the spectra of samples containing goethite—and the spectra are provided in Figure 1, with hyperfine parameters presented in Table 1 (sextet A + sextet B for magnetite, sextet C or a hyperfine field distribution/B_hf_ Dist. for goethite, and a doublet for ferrihydrite). The uncertainties of the fitted parameters, subspectral areas, and linewidths have been included in the new table. It can be observed that goethite amounts, if any, are not small at all, justifying the point raised by the questioner. It is also worth noting the (large) linewidths found for the goethite discrete sextets, indicating some degree of hyperfine field distribution.

Finally, we emphasize that the Mössbauer analysis—in spite of the recognized problems in the fit methodology—accomplished its role as a fingerprint technique, since it confirmed the presence of magnetite, goethite, and ferrihydrite phases. In conclusion, after correcting the fits and fixing the table of hyperfine parameters, the main conclusions of the paper are exactly the same as before.

## Figures and Tables

**Figure 1:**
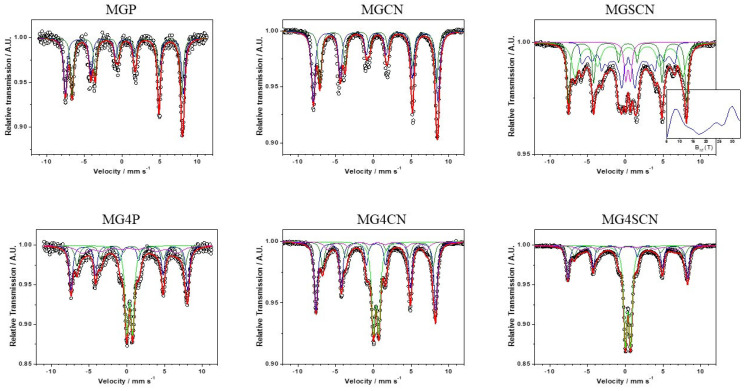
Mössbauer spectra of all magnetite samples.

**Table 1:** Mössbauer hyperfine parameters of all samples and iron mineral correspondence.

Sample	Mineral	Sub Spectrum	Γ/ mm s^−1^	IS/ mm s^−1^	QS/ mm s^−1^	Bhf/ T	A/ %
MGP	Magnetite	Sext A	0.52	0.34	−0.08	48.5	52.2
		Sext B	0.46	0.65	−0.01	45.1	47.8
MGP4	Magnetite	Sext A	0.53	0.33	0.00	47.9	32.0
		Sext B	0.64	0.62	−0.12	44.5	17.8
	Ferrihydrite	Doublet	0.57	0.37	0.78	-----	31.0
	Goethite	Sext C	1.99	0.35 *	0.07 *	39.0 *	19.2
MGCN4	Magnetite	Sext A	0.50	0.33	−0.02	49.2	30.0
		Sext B	0.67	0.55	0.08	45.3	12.8
	Ferrihydrite	Doublet	0.52	0.36	0.69	-----	42.8
	Goethite	Sext C	1.60 *	0.36	0.07	39.0	14.4
MGSCN4	Magnetite	Sext A	0.50	0.33	−0.02	49.2	30.2
		Sext B	0.70	0.51	0.07	45.2	13.4
	Ferrihydrite	Doublet	0.52	0.36	0.69	-----	42.9
	Goethite	Sext C	1.60 *	0.35 *	0.08	38.6	13.5
MGCN	Magnetite	Sext A	0.52	0.33	−0.08	48.5	59.7
		Sext B	0.48	0.65	−0.09	45.4	40.3
MGSCN	Magnetite	Sext A	0.47	0.32	−0.04	48.5	23.7
		Sext B	1.09	0.47	0.02	44.2	30.5
	Ferrihydrite	Doublet	0.51	0.37	0.63	-----	7.3
	Goethite	Dist.	0.30 *	0.39	0.00 *	25.1	38.5

* Fixed parameters. (Γ—Line width; IS—Isomer shift; QS—Quadrupole splitting; B_hf_—Magnetic hyperfine field; A—Subspectrum area).

## References

[1] Nayak P.K. (2021). Comment on Samulewski et al. Magnetite Synthesis in the Presence of Cyanide or Thiocyanate under Prebiotic Chemistry Conditions. *Life* 2020, *10*, 34. Life.

[2] Samulewski R.B., Gonçalves J.M., Urbano A., da Costa A.C.S., Ivashita F.F., Paesano A., Zaia D.A.M. (2020). Magnetite Synthesis in the Presence of Cyanide or Thiocyanate under Prebiotic Chemistry Conditions. Life.

